# Chemical Plants Remain Vulnerable to Terrorists: A Call to Action

**DOI:** 10.1289/ehp.8762

**Published:** 2006-04-27

**Authors:** Tobi Mae Lippin, Thomas H. McQuiston, Kristin Bradley-Bull, Toshiba Burns-Johnson, Linda Cook, Michael L. Gill, Donna Howard, Thomas A. Seymour, Doug Stephens, Brian K. Williams

**Affiliations:** 1 New Perspectives Consulting Group Inc., Durham, North Carolina, USA; 2 United Steel, Paper and Forestry, Rubber, Manufacturing, Energy, Allied Industrial and Service Workers International Union (USW), Tony Mazzocchi Center for Safety, Health and Environmental Education, Pittsburgh, Pennsylvania, USA; 3 USW, Local Union 500689, Piketon, Ohio, USA; 4 USW, Local Union 5, Martinez, California, USA; 5 USW, Local Union 7-0706, Indianapolis, Indiana USA

**Keywords:** anti-terrorism, chemical plant security, emergency response, hazardous materials, prevention

## Abstract

U.S. chemical plants currently have potentially catastrophic vulnerabilities as terrorist targets. The possible consequences of these vulnerabilities echo from the tragedies of the Bhopal incident in 1984 to the terrorist attacks on 11 September 2001 and, most recently, Hurricanes Katrina and Rita. Findings from a 2004 nationwide participatory research study of 125 local union leaders at sites with very large volumes of highly hazardous chemicals suggest that voluntary efforts to achieve chemical plant security are not succeeding. Study respondents reported that companies had only infrequently taken actions that are most effective in preventing or in preparing to respond to a terrorist threat. In addition, companies reportedly often failed to involve key stakeholders, including workers, local unions, and the surrounding communities, in these efforts. The environmental health community thus has an opportunity to play a key role in advocating for and supporting improvements in prevention of and preparation for terrorist attacks. Policy-level recommendations to redress chemical site vulnerabilities and the related ongoing threats to the nation’s security are as follows: *a*) specify detailed requirements for chemical site assessment and security; *b*) mandate audit inspections supported by significant penalties for cases of noncompliance; *c*) require progress toward achieving inherently safer processes, including the minimizing of storage of highly hazardous chemicals; *d*) examine and require additional effective actions in prevention, emergency preparedness, and response and remediation; *e*) mandate and fund the upgrading of emergency communication systems; and *f*) involve workers and community members in plan creation and equip and prepare them to prevent and respond effectively to an incident.

The nation’s chemical infrastructure is at risk of terrorist attack (U.S. Department of Justice 2000). The vulnerabilities of chemical-related industries, although recognized before 11 September 2001, became dramatically more pressing that day after terrorists demonstrated the capacity for catastrophic strikes against key U.S. targets. In 2003, the Homeland Security Advisory System issued alerts that identified the U.S. nuclear and chemical manufacturing infrastructure as potential terrorist targets (National Infrastructure Protection Center 2003a, 2003b). That same year, the General Accounting Office [now the Government Accountability Office (GAO)] identified chemical facilities as potentially attractive targets threatening nearby population centers (GAO 2003a). The GAO linked these potential vulnerabilities to more than 15,000 sites across the United States identified by the U.S. Environmental Protection Agency (EPA) as Risk Management Program (RMP) sites with large volumes of highly hazardous chemicals.

The GAO (2003a) showed that 123 chemical facilities have worst-case scenarios involving more than a million people in the surrounding area at risk of exposure to a cloud of toxic gas if a release occurred. Estimates show that 700 sites could put 100,000 people at risk, and approximately 3,000 sites could put 10,000 people at risk.

RMP worst-case estimates, although valuable, have limitations for assessing the possible consequences of attacks on chemical facilities. First, releases caused by intentional acts may differ in size, scope, and severity from accidental releases from a single vessel or process line (Belke 2000; GAO 2003a). Second, RMP consequence analyses involve off-site communities rather than on-site populations. Third, people estimated to be directly affected by a toxic release would be limited to those in the path of the toxic plume, not necessarily the entire population within a theoretical zone of vulnerability.

Although threats to the nation’s chemical infrastructure loom large, limited data are available to gauge progress in prevention, pre-paredness, or response (Falkenrath 2005). This mirrors the lack of useful data to assess industry progress in the prevention of serious chemical accidents (Hood 2004; Mary Kay O’Connor Process Safety Center 2002a; Poje 2005).

Facilities with large volumes of highly hazardous chemicals employ multiple layers of protection for potential chemical releases, fires, and explosions. These layers include security measures to control and limit access, containment and other mitigation systems, automatic warnings and alarms, and emergency response. In general, a hierarchy that places primary emphasis on design and engineering solutions governs the potential effectiveness of each layer of protection (Reason 1999, 2000; Renner 2004).

Since September 11, there have been repeated demonstrations of the potential for terrorists to skirt security and gain access to chemical facilities (CBS News 2003; Prine 2002, 2003). Accordingly, some policy proponents seek to expand the focus from perimeter security to more effective layers of protection that would first make chemical facilities less vulnerable targets and then, if attacked, limit the consequences (Corzine 2005).

A major strategy for this expanded focus is the concept of “inherently safer” technologies [Kahn and Amyotte 2003; Organisation for Economic Co-operation and Development (OECD) 2003a]. These technologies aim to eliminate or minimize the potential for catastrophic events by designing hazards out of process systems (Kletz 1996). Inherently safer design strategies include *a*) substituting highly hazardous substances with less hazardous ones; *b*) minimizing levels of hazardous materials and energy; *c*) moderating hazards with the use of alternative forms of materials; and *d*) reducing unnecessary systems complexity to increase process controllability (Crowl 1996). Such improvements would limit not only the desirability of sites as terrorist targets but also the consequences of such an attack. Further, inherently safer improvements would reduce overall day-to-day risks of an unintentional incident affecting the plant, the community, and the environment.

Despite the consensus about chemical site vulnerability, there is evidence from the U.S. Chemical Safety and Hazard Investigation Board (CSB) that government, companies, workers, responders, and the public are not adequately prepared for unintentional incidents (Merritt 2005). These findings from the CSB provide valuable insight into possible intentional incidents. An independent task force sponsored by the Council on Foreign Relations concluded that the nation’s emergency responders were “drastically under-funded and dangerously unprepared” for another major terrorist attack, especially “one involving chemical, biological, radiological, or nuclear agents” (Rudman et al. 2003, p. 7).b The GAO (2003b) found similar deficiencies in hospital preparedness.

These deficiencies are in contrast to the comprehensive needs in the areas of prevention, preparedness, response, recovery, and mitigation enunciated for government and the private sector in the National Incident Management System [U.S. Department of Homeland Security (DHS) 2004b] and the related National Response Plan (DHS 2004a). Most recently, the GAO (2006) reported that *a*) the chemical industry still “faces challenges in preparing against terrorism” (p. 5); *b*) despite its voluntary programs, “the extent to which individual companies across the industry are addressing security issues is unclear” (p. 57); *c*) “DHS cannot ensure that all high-risk facilities are assessing their vulnerability to terrorist attacks and taking corrective actions, where necessary” (p. 6); and *d*) DHS has concluded that “its existing authorities do not permit it to effectively regulate the industry, and that the Congress should enact federal requirements for chemical facilities” (pp. 6–7).

At the community level, findings from a survey of households within a 1-mi radius of sites with large volumes of highly hazardous chemicals (i.e., RMP sites) also showed a lack of preparedness (Mary Kay O’Connor Process Safety Center 2002b). Among households defined as living near chemical facilities that are considered at high risk for a release, only one-fourth were aware of the facilities posing the risk. Similarly, less than one-third of this high-risk group believed that community members were informed (very well or adequately) about where to get information in a chemical emergency.

The DHS’s *Interim National Infrastructure Protection Plan* (DHS 2005) recognizes the importance of participation of all stakeholders. Similarly, a review of 239 published case studies in environmental decision making concludes that involved stakeholders contribute new ideas and analysis and improve decisions (Beierle 2002). Following the guidance of the U.S. Clean Air Act (U.S. EPA 1990), the New Jersey Toxic Catastrophe Prevention Act (State of New Jersey 2005) has also validated the importance of worker involvement in chemical plant safety by recognizing the right of employees and their representatives to participate in facility inspections and investigations.

## Legislative and regulatory approaches

To address these many documented risks and deficiencies, in April 2005 the U.S. Senate Committee on Homeland Security and Governmental Affairs (2005) began a series of hearings on the security of the U.S. chemical industry. During these hearings, Stephenson (2005) of the GAO stated that both the federal government and the chemical industry have taken some necessary steps but that the nation needs to take further action. Stephan (2005), representing the DHS, provided similar testimony before this same Senate committee, concluding that the current “patchwork” of authority does not form the basis for effective regulation. In response to a 2003 report (GAO 2003a), the DHS noted that voluntary efforts were inadequate to address possible threats and that all RMP sites “should be required to perform comprehensive vulnerability assessments and take actions to reduce vulnerabilities” (Stephenson 2005, p. 5).

## Participatory Research Study

In an effort to determine whether disaster prevention and preparedness had improved since September 11, a research team—originally from the Paper, Allied-Industrial, Chemical and Energy Workers International Union (PACE) and now, postmerger, part of the United Steel, Paper and Forestry, Rubber, Manufacturing, Energy, Allied Industrial and Service Workers International Union (USW)—conducted a national study of RMP sites (PACE 2004) using a participatory research approach (Israel et al. 1994; McQuiston 2000). The participatory research team included USW rank and file workers and staff as well as education and evaluation consultants (PACE 2004). The team designed the survey instrument to assess union leaders’ perceptions of activities since September 11, including company actions to improve prevention and emergency response and their effectiveness, and the involvement in these issues by the local union, hourly workers, and the community. Surveys were mailed between March and June 2004 to union leaders at 189 sites that, at the time of the study, were PACE-represented(now USW-represented) RMP sites. The response rate was 70% and included 133 sites in 37 states (PACE 2004).

Findings of the study (PACE 2004) were limited to the 95% of sites (*n* = 125) determined to be at greatest risk based on each respondents’ assessment that the site had quantities of hazardous materials large enough to cause a catastrophic event on-site if those materials were involved in a fire, explosion, or other release. Notably, 80% of these sites (*n* = 100) also reported quantities of hazardous materials large enough to cause a catastrophic event in the areas surrounding the plant.

## Data Highlights

### Respondent site profile

The analysis (PACE 2004) included responses from union leaders at 40 chemical plants (32% of sites), 32 primary paper mills (26%), 30 oil refineries (24%), and 23 other types of industries (18%). Regarding the size of the workforces at these sites, 14 (11%) had ≥ 1,000 employees, 31 (25%) had 500–999 employees, 55 (44%) had 100–499 employees, and 25 (20%) had < 100 employees.

### Company actions

In the PACE study (PACE 2004), respondents were asked about a variety of possible company actions taken since September 11 aimed at preventing a catastrophic event caused by a terrorist attack. By far, the most frequently reported preventative improvements were systems to guard the plant (73%), assessment of site vulnerabilities (66%), and reassessment of worksite security (64%) (Table 1). In contrast, the reduction of volumes of hazardous substances—a step to make sites inherently safer—was one of the least frequently reported actions (17%), along with design and engineering changes to strengthen chemical containment (17%) and to improve the siting of chemicals to less vulnerable areas (14%). Thus, companies reportedly tended to take fewer steps that are more effective in addressing fundamental vulnerabilities key to preventing an event.

The survey also queried respondents about company actions to prepare them to respond to a terrorist incident (PACE 2004). Respondents most frequently reported that companies provided employee emergency response training (68%) and conducted emergency response drills (59%) (Table 1). According to respondents, less than one-third of companies had updated emergency shutdown procedures for equipment (30%), informed hospitals and others about potential health threats from plant-specific exposures (23%), updated their emergency response plan for the community (21%), or added procedures to inform the community about an emergency (15%). Thus, the reportedly least frequently taken actions were those that might also help both on-site workers and surrounding communities better address an event perpetrated at the site.

In addition, respondents provided detail about the company training noted above. Overall, it was reportedly rare that companies trained a majority of their workforces in these issues. Fifteen percent or fewer of the respondents reported the provision of prevention and response preparedness training to greater than one-half of their site’s work-force since September 11 (PACE 2004). A sizable proportion of respondents reported no employee training in preventing (34%) or responding to (28%) an event. Moreover, 74% of respondents reported that union employees at these sites needed additional training of this type.

### Effectiveness of company actions

**Figure 1:**
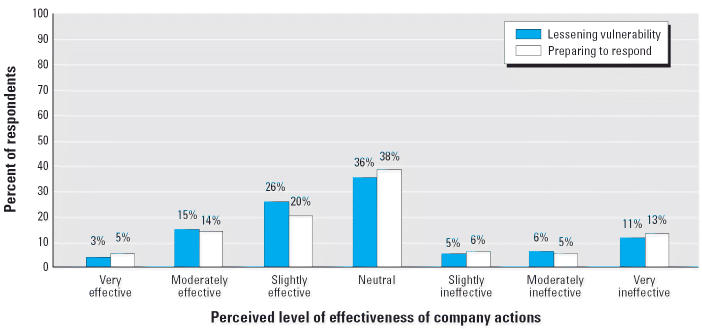
Perceived effectiveness of company actions in lessening vulnerability to and in preparing to respond to a terrorist attack based on the percentage of respondents choosing each survey answer. Bars represent the reported effectiveness of company actions in either lessening vulnerability of the worksite or preparing the worksite to respond to a catastrophic event caused by a terrorist attack. Answers were in response to survey question 6: Overall, since September 11, how effective have the actions taken by the company been in lessening the vulnerability of your worksite to a catastrophic event caused by [a terrorist attack]? (*n* = 124, 0.8% missing); and survey question 8: Overall, since September 11, how effective have the actions taken by the company been in preparing your worksite for a catastrophic event caused by [a terrorist attack]? (*n* = 125, 0.0% missing). Percentages may not add up to 100% because of rounding.

Only 18% of respondents reported that their companies’ prevention actions had been more than “slightly effective” in lessening their plant’s vulnerability to terrorist attack (Figure 1). The study found similar results for the effectiveness of companies’ actions to prepare their work-sites to respond to a potentially catastrophic event.

### Collaboration among key parties

Respondents also reported that it was rare that companies worked with the local union, hourly workers, or the community regarding related prevention or response planning or action (PACE 2004). Twenty-eight percent or fewer respondents reported some type of involvement of the local union, hourly workers, or the community. Typically, the reported involvement included the company informing these groups rather than engaging them in assessing the situation or making recommendations. Almost two-thirds of respondents reported that they were unaware of whether the company worked with the community in these ways.

## Study Limitations

The study’s findings (PACE 2004) provide respondent perceptions rather than independent assessments of employer actions. Respondents also frequently selected “don’t know” for items related to company actions. To our knowledge, The PACE survey (PACE 2004) is the first survey conducted among local union leaders on this topic. Therefore, there is no basis for knowing if this is typical. However, the “don’t know” responses (Table 1) do provide important data about the level of hourly worker and local union leader involvement in these issues.

In addition, security measures have limited the public’s access to the RMP data used in site selection, making it possible that some PACE-represented sites included were not RMP sites and vice versa. Further, the design of this study precludes generalizability to all former PACE-or current USW-represented workplaces or industrial sectors or to other RMP sites.

## Conclusions and Recommendations

The PACE study (PACE 2004) shows that primarily voluntary initiatives since September 11 have not yielded a level of progress on chemical plant security commensurate with the potential catastrophic consequences posed by terrorist threats. This is especially remarkable given the potential consequences of a terrorist attack, coupled with the devastating lessons of Bhopal, September 11, and most recently, Hurricanes Katrina and Rita. Overall, the levels of activity and achievement reported in the PACE study (PACE 2004) reinforce recent congressional testimony that voluntary measures alone are insufficient. This is consistent with the contention of Richard Falkenrath, former Deputy Assistant to President George W. Bush and Homeland Security Advisor, that

It is a fallacy to think that profit-maximizing corporations in a trade as inherently dangerous as the manufacture and shipment of TIH [toxic inhalation hazard] chemicals will ever voluntarily provide the level of security that is appropriate given the larger external risk to society as a whole. (Falkenrath 2005, p. 13)

The United States has already lost much precious time in preparing for the possibility of a catastrophic chemical release and urgently needs laws and regulations capable of driving dramatic improvements in chemical plant security. If the nation is to reach its safety and security goals of protecting communities and workers, these legislative and regulatory initiatives will need to be enforceable and to provide sufficient guidance to ensure the application of adequate resources. These initiatives will also require both ongoing evaluation—guided by tools such as the OECD’s activities and outcome indicators (OECD 2003b)—and reporting of progress to both Congress and the public. Fortunately, the environmental health community’s broad range of expertise makes it uniquely qualified to support these initiatives by advocating for and developing policy, providing scientific consultation, evaluating results, and informing and engaging key stakeholders. Below we provide recommendations for legislative and regulatory action to address these issues.

### Recommendation 1: Specify detailed requirements for chemical site assessment and security

Although survey respondents indicated that most of the facilities had conducted vulnerability assessment and security enhancements since September 11, their responses also suggest that many sites still have not taken these initial steps. This is consistent with news reports of the ease with which outsiders have gained unfettered access to reportedly secured chemical plants (CBS News 2003; Prine 2002, 2003).

### Recommendation 2: Mandate audit inspections supported by significant penalties for cases of noncompliance

Detailed requirements (Recommendation 1) need to be coupled with audit inspections by appropriate government agencies and certain and swift enforcement with severe penalties for noncompliance (McQuiston et al. 1998). Rigorous, uniform enforcement of mandatory requirements will help ensure that unresponsive companies do not gain competitive advantage over those that comply.

### Recommendation 3: Require progress toward achieving inherently safer processes, including the minimizing of storage of highly hazardous chemicals

Improvements in perimeter security can never ensure a site’s safety from terrorist attacks and can divert scarce resources away from overall plant safety. The most effective ways to prevent RMP sites from becoming weapons of mass destruction are to eliminate or drastically reduce the substances that, if released into the environment, could kill or harm thousands of workers and community members.

The value and potential of chemical substitution, a primary means for creating inherently safer processes, have been achieved in many cases. For example, water treatment plants have replaced chlorine gas with less hazardous chemicals, and technologies exist to replace large quantities of hydrofluoric acid at oil refineries (Purvis and Herman 2005). Offering further evidence of the viability of inherently safer approaches, approximately one in six sites in this study reported having acted since September 11 to reduce their volumes of hazardous substances. Industry must be required to apply these approaches as broadly as possible.

### Recommendation 4: Examine and require additional effective actions in prevention, emergency preparedness, and response and remediation

Certainly, in the short term, not all companies will find and apply inherently safer approaches universally. Therefore, additional requirements that help bridge this gap will create safer chemical sites. For example, provisions should ensure that reactive materials are adequately isolated from each other; that plant infrastructure is hardened against attack; and that mitigation systems sufficient to suppress, neutralize, and contain hazards are ready should vessels be breached.

### Recommendation 5: Mandate and fund the upgrading of emergency communication systems

There may be limited if any advanced warning of a chemical attack. Effective emergency communication systems must be operative if facilities and communities are to respond immediately and effectively. The government must ensure that the private sector finally upgrades interoperable emergency communication systems that were proven dysfunctional on September 11 and again during Hurricane Katrina. These requirements will help ensure the viability of both the National Incident Management System (DHS 2004a) and the National Response Plan (DHS 2004b).

### Recommendation 6: Involve workers and community members in plan creation and equip and prepare them to prevent and respond effectively to an incident

Chemical site employees, who will be at ground zero in the event of a terrorist attack, are the primary stakeholders in plant security issues. Further, these employees’ collective expertise is vital to improving safety and security for themselves and their communities. Indeed, the American Chemistry Council’s commitment to safety and performance lists “operators,” who are typically hourly employees, along with chemists and engineers as “experts in the business of managing and reducing risks associated with making chemicals” (American Chemistry Council 2002, p. 6). Similarly, the Occupational Safety and Health Process Safety Management Standard requires employers to

consult with employees and their representatives on the conduct and development of process hazards analyses and on the development of the other elements of process safety management. [Occupational Safety and Health Administration (OSHA) 1992]

Also, community members, including local government officials and emergency responders, will be essential players in this planning and implementation effort. Effective involvement for all parties requires a monumental training initiative targeting site workers, emergency responders, remediation workers, and communities. The National Institute of Environmental Health Sciences Worker Education and Training Program (Research Triangle Park, NC) offers a highly successful model program.

Fortunately, some of the policy instruments needed to operationalize and enforce these recommendations are already in place. Congress needs only to mandate adaptations of the existing OSHA standards and U.S. EPA regulations to terrorist threats (OSHA 1992; U.S. EPA 1996). The DHS, OSHA, and the U.S. EPA should each enforce the parts of legislation that focus on its area of expertise.

In conclusion, as policy makers grapple with tangible ways to enhance U.S. security against possible terrorist threats, these recommendations represent a rare opportunity to advance this mission at a pace appropriate for looming dangers. Chemical site employees, surrounding communities, and the nation deserve no less.

## Figures and Tables

**Table 1:** Company actions to prevent and prepare to respond to a catastrophic event.

Was action taken?	Yes (%)	No (%)	Don’t know (%)
Prevention			
1. Improved systems to guard and secure the plant^a^,^b^	72.8	23.2	4.0
2. Assessed vulnerabilities^c^,^d^	66.4	12.0	21.6
3. Reassessed worksite security^a^,^b^	64.0	19.2	16.8
4. Updated warning systems^b^,^c^	38.4	48.8	12.8
5. Improved training and procedures to prevent possible terrorist attacks^b^,^c^	37.6	54.4	8.0
6. Improved containment of potential hazards^b^,^c^	33.6	50.4	16.0
7. Reduced volumes of hazardous substances^b^,^c^	16.8	60.0	23.2
8. Strengthened plant vessels, tanks, piping, or other structures^b^,^c^	16.8	65.6	17.6
9. Improved the siting of hazardous substances or processes to less vulnerable locations^b^,^c^	13.6	68.8	17.6
Preparation to respond			
10. Provided emergency response training to employees within the past 12 months^e^,^f^	67.5	26.0	6.5
11. Conducted emergency response drills for the plant site^e^,^g^	58.9	35.5	5.6
12. Updated emergency response plan for the facility^e^,^g^	46.8	33.1	20.2
13. Informed local fire and police departments, HazMat teams, etc., about potential plant-specific hazards^e^,^f^	45.5	14.6	39.8
14. Put in place additional procedures to inform employees of an emergency (e.g., alarms, public address system)^e^,^g^	41.9	50.8	7.3
15. Improved quality and availability of personal protective equipment^c^,^g^	30.4	57.6	12.0
16. Updated shutdown procedures for critical equipment in an emergency^e^,^g^	29.8	41.1	29.0
17. Informed local hospitals, health departments, emergency medical personnel, etc., about the potential health threats from plant-specific exposures^e^,^g^	23.4	20.2	56.5
18. Updated emergency response plan for the community^e^,^g^	21.0	33.9	45.2
19. Put in place additional procedures to inform the community about an emergency (e.g., alarms, public address system)^e^,^g^	15.3	45.2	39.5

Percentages may not add up to 100% because of rounding. All data are from PACE (2004).

a Responses to survey question 5 (items 1 and 3): Since September 11, has the company at your worksite done any of the following related to plant security in the face of new terrorist threats?

b Items 1 and 3–9, *n* = 125, 0.0% missing.

c Responses to survey question 4 (items 2, 4–9, and 15): Since September 11, has the company at your worksite taken any of the following actions to prevent a catastrophic event caused by a terrorist attack?

d Item 2, *n* = 124, 0.8% missing.

e Responses to survey question 7 (items 10–14,16–19): Since September 11, has the company at your worksite taken any of the following actions to be better prepared to respond to a catastrophic event that was caused by a possible terrorist attack?

f Items 10 and 13, *n* = 123, 1.6% missing.

g Items 11–12 and 14–19, *n* = 124, 0.8% missing.
